# Elevated N-terminal pro C-type natriuretic peptide is associated with mortality in patients undergoing transcatheter aortic valve replacement

**DOI:** 10.1186/s12872-022-02615-8

**Published:** 2022-04-12

**Authors:** Po Hu, Han Chen, Li-Han Wang, Ju-Bo Jiang, Jia-Min Li, Meng-Yao Tang, Yu-Chao Guo, Qi-Feng Zhu, Zhao-Xia Pu, Xin-Ping Lin, Stella Ng, Xian-Bao Liu, Jian-An Wang

**Affiliations:** 1 Department of Cardiology, The Second Affiliated Hospital, School of Medicine, Zhejiang University, Hangzhou, 310009 Zhejiang China; 2 Department of Echocardiography, The Second Affiliated Hospital, School of Medicine, Zhejiang University, Hangzhou, 310009 Zhejiang China; 3 Cardiovascular Key Laboratory of Zhejiang Province, Hangzhou, 310009 Zhejiang China; 4 Renal Division, Brigham and Women’s Hospital, Boston, MA USA; 5 Division of Nephrology, Department of Medicine, Massachusetts General Hospital, Boston, MA USA

**Keywords:** Transcatheter aortic valve replacement, Mortality, Natriuretic peptides, NT-proCNP

## Abstract

**Background:**

Unlike N-terminal pro-B-type natriuretic peptide (NT-proBNP), which have been extensively studied, little is known about the role of N-terminal pro-C-type natriuretic peptide (NT-proCNP) for predicting survival post transcatheter aortic valve replacement (TAVR).

**Methods:**

A total of 309 patients were included in the analysis. Patients were grouped into quartiles (Q1–4) according to the baseline NT-proCNP value. Blood for NT-proCNP analysis was obtained prior to TAVR procedure. The primary endpoint was mortality after a median follow-up of 32 months. Multivariable Cox proportional hazards regression models analyzed prognostic factors. The predictive capability was compared between NT-proBNP and NT-proCNP using receiver operator curve (ROC) analysis.

**Results:**

A total of 309 subjects with the mean age of 76.8 ± 6.3 years, among whom 58.6% were male, were included in the analysis. A total of 58 (18.8%) patients died during follow-up. Cox multivariable analyses indicated society of thoracic surgeons (STS)-score was a strong independent predictor for mortality (hazard ratio (HR) 1.08, 95% confidential interval (CI) 1.05–1.12, *P* < 0.001). Elevated NT-proCNP was associated with a higher risk of cardiovascular mortality (HR 1.02, 95% CI 1.00–1.03, *P* = 0.025) and All-cause mortality (HR 1.01, 95% CI 1.00–1.03, *P* = 0.027), whereas NT-proBNP showed a small effect size on mortality. ROC analysis indicated that NT-proCNP was superior to NT-proBNP for TAVR risk evaluation in patients with left ventricular ejection fraction (LVEF) < 50% [(Area under the curve (AUC)-values of 0.79 (0.69; 0.87) vs. 0.59 (0.48; 0.69), *P* = 0.0453].

**Conclusions:**

NT-proCNP and STS-Score were the independent prognostic factors of mortality among TAVR patients. Furthermore, NT-proCNP was superior to NT-proBNP for TAVR risk evaluation in patients with LVEF < 50%.

*Trial registration* NCT02803294, 16/06/2016.

**Supplementary Information:**

The online version contains supplementary material available at 10.1186/s12872-022-02615-8.

## Introduction

Unlike A -type natriuretic peptide (ANP) and B -type natriuretic peptide(BNP), which have been extensively studied, C-type natriuretic peptide (CNP)'s role in cardiovascular disease has not yet been fully understood. CNP is an endothelial product that is released as a protective mechanism in response to cardiovascular disease [1]. Several experimental studies have demonstrated that CNP could suppress myocardial fibrosis, collagen synthesis, and cardiac fibroblast proliferation [2–5]. Community-based study has reported that CNP could have prognostic value in predicting future risk of cardiovascular comorbidities and left ventricular dysfunction [1]. CNP has also been implicated in the pathophysiology of heart failure [6] and aortic valve calcification [7].

CNP has been reported to circulate at very low concentrations in adults and to have a short half-life due to rapid degradation [8, 9]. NT-proCNP is derived from the processing of proCNP to CNP and co-secreted with CNP. NT-proCNP is considered a more reliable marker that can be more easily measured than CNP [10, 11]. In heart failure, the NT-proCNP concentration has been associated with clinical severity [12]. This proposed relationship has been supported by the COACH [13] sub-study, which has demonstrated that NT-proCNP is a strong predictive marker for outcome in patients with heart failure [14].

Aortic stenosis (AS) is a common valve disease in the elderly population that comprises one of the major cardiovascular morbidities among older patients [15, 16]. Persistent pressure overload due to AS promotes left ventricular remodeling and results in an increased risk of heart failure [17]. TAVR has emerged as an alternative to conventional aortic valve replacement for patients with aortic stenosis [18, 19]. Although NT-proCNP has a prognostic value in patients with heart failure, little is known about the role of NT-proCNP for predicting survival post-TAVR. The purpose of the current study was to assess whether baseline NT-proCNP is associated with mortality in patients undergoing TAVR.

## Material and methods

### Study design and population

The analysis cohort comprised consecutive patients undergoing TAVR between March 2013 and November 2018. This cohort has been approved by the local ethic committee in 2013 and has been registrated at www.clinicaltrials.gov (identifier NCT02803294, 16/06/2016). The patients were followed up until death or until the last follow-up on 31 January 2020. The study aimed to evaluate the safety and effectiveness of transcatheter aortic valve replacement in the Chinese population. The TAVR procedure and the devices used have been described in detail previously [20]. Briefly, experienced operators in our center with an established multidisciplinary TAVR program performed all procedures. Most patients were implanted with self-expanding valves, followed by balloon-expandable Edwards SAPIEN XT or SAPIEN 3 valves or mechanically expandable Lotus valve. Self-expanding valves used in this registry included the CoreValve (Medtronic, Minneapolis, Minnesota), VenusA-Valve (Venus Medtech, Hangzhou, China), VitaFlow (Microport, Shanghai, China), and Taurus One-Valve (Peijia Medical, Suzhou, China). A large proportion of patients were treated with 100 mg of aspirin daily indefinitely and 75 mg of clopidogrel for at least 3 months, when anticoagulation treatment was indicated, patients received warfarin or new oral anticoagulants. This study was approved by the local ethics committee and conducted in accordance with the Declaration of Helsinki. All patients provided written informed consent.

### Laboratory assessments

Laboratory measurements were done at our institution’s core facility. Routine biomarker analysis was obtained prior to discharge. Blood for NT-proCNP analysis was collected in the fasting state in EDTA tubes, placed on ice, then centrifuged at 5000 rpm for 10 min at 20 °C and the plasma was stored at − 80 °C. According to the manufacturer's instructions, the circulating levels of NT-proCNP were measured with a commercial ELISA kit (Enzo Biochem, New York, USA) at room temperature. Each well was briefly coated with 50 µL assay buffer, and 20 µL standard or serum sample was added in duplicate and incubated for 20 min. Then, 50 µL conjugate was added and incubated for 3 h, followed by 100 µL substrate and incubated for 30 min. Then, 50 µL stop solution was applied at the end of incubation, and the absorbance was immediately measured at 450 nm with a reference at 630 nm.

### Clinical data and endpoints

Patients underwent a standard screening algorithm including transthoracic echocardiograms and enhanced cardiac Computed Tomography (CT). Aortic annulus size was measured by cardiac CT in 3mensio soft-ware (3mensio Medical Imaging BV, Bilthoven, the Netherlands). The following clinical information was collected: age, gender, body mass index (BMI), New York Heart Association (NYHA) classification, comorbidities [i.e., hypertension, diabetes mellitus (DM), dyslipidemia, peripheral vascular disease, cerebrovascular disease, chronic obstructive pulmonary disease (COPD) and chronic kidney disease], and STS score. Clinical data, electrocardiogram, transthoracic echocardiograms were obtained at baseline, hospital discharge, 30 days, 1 year, and annually after that. The primary endpoint was cumulative mortality, including all-cause mortality and cardiovascular mortality. The secondary endpoint implied major adverse cardiac events (MACE), defined as a composite of all-cause mortality, myocardial infarction, stroke, permanent pacemaker implantation, new-onset atrial fibrillation, and reoperation for prosthetic valve degeneration.

### Statistical analysis

Continuous variables are presented as mean ± standard deviation (for normally distributed variables), or median, 25th, and 75th percentile (for non-normally distributed variables). Categorical variables are reported as percentages. Baseline characteristics were grouped into quartiles (Q1–4) according to the baseline NT-proCNP. The intergroup differences were evaluated by Kruskal–Wallis test, one-way ANOVA test, or χ^2^ tests where appropriate. The correlations between laboratory parameters and NT-proCNP levels were analyzed. Correlations were performed using Pearson’s correlation coefficient.

Performance of three models predicting cumulative mortality in the study population was assessed and compared for the following three logistic regression models: model 1 (NT-ProBNP, NT-ProCNP or STS score only), model2 (NT-ProBNP + STS-score), and model 3 (NT-ProCNP + STS-score). The area under the receiver operating characteristic curve, cut off value and Hosmer–Lemeshow statistics were estimated. Kaplan–Meier estimates were used to estimate survival curves and the log-rank test to test differences between groups. Cox proportional hazards multiple regression models were used to calculate the predictive value of NT-proCNP/NT-ProBNP for primary endpoints, after adjusting for age, gender, BMI, LVEF, hypertension, creatinine, STS score, Access for TAVR, prosthetic valve of TAVR. A two‑sided *P* < 0.05 was considered as statistically significant. The SPSS for Windows version 22.0 (SPSS Inc., Chicago, IL, USA) was used for the statistical analyses. The MedCalc statistical software was used for the comparison of ROC curves.

## Results

### Patient population and baseline characteristics

The baseline characteristics by quartiles of plasma NT-proCNP of the 309 subjects are shown in Table 1. The mean age of the 309 subjects was 76.8 ± 6.3 years, and 58.6% were male. Mean LVEF was 53.8 ± 13.6%, and 89.3% were in NYHA class III or IV. The most common comorbidity was hypertension, followed by dyslipidemia, COPD, and DM. Most patients were treated with trans-femoral TAVR (93.2%), followed by trans-apical (2.3%), trans-carotid (2.3%), trans-aortic (1.6%), and trans-subclavian TAVR (0.6%). A large proportion of patients adopted self-expandable valves (84.8%), followed by balloon-expandable Edwards SAPIEN XT or SAPIEN 3 valves (8.1%) or mechanically expandable Lotus valve (7.1%). The median level of NT-proCNP was 30.10 (IQR 23.59–41.05) pmol/L. Higher NT-proCNP levels were significantly associated with higher levels of NT-proBNP and STS scores. Lower LVEF was more common among patients with a higher level of NT-proCNP, while rates of NYHA class III or IV were similar between the four groups. There were no differences between the four groups in mean gradient, maximum velocity, and aortic valve area. The mean estimated glomerular filtration rate (eGFR) was 70.7 ± 22.9 mL/min/1.73 m^2^, and higher NT-proCNP values were significantly associated with the declined renal function (*P* < 0.001). A significant correlation was found between NT-proCNP and eGFR, whereas the association was not strong enough (r =  − 0.47, *P* < 0.001). Moreover, there was a strong positive association between NT-proCNP and creatinine (r = 0.79; *P* < 0.001).

**Table 1:** Baseline characteristics of all patients, stratified into quartiles of NT-pro CNP (pmol/L)

Variable	All (*n* = 309)	Q1 (*n* = 78)	Q2 (*n* = 77)	Q3 (*n* = 77)	Q4 (*n* = 77)	*P* value
NT-proCNP, min–max (pmol/L)	12.36–360.95	12.36–23.61	23.73–30.05	30.08–41.03	41.06–360.95	–
Age (years)	76.8 ± 6.3	76.3 ± 6.3	77.5 ± 5.4	76.9 ± 6.7	77.8 ± 7.2	0.20
Male sex	58.6 (181/309)	50.0 (39/78)	57.1 (44/77)	68.8 (53/77)	58.4 (45/77)	0.12
Body mass index (kg/m^2^)	22.6 ± 3.5	23.5 ± 3.7	23.0 ± 3.4	22.3 ± 3.1	21.8 ± 3.1	0.03
STS score	7.2 ± 5.7	6.0 ± 3.2	5.8 ± 3.0	7.5 ± 5.1	8.9 ± 6.4	0.001
TTE data						
Mean gradient, mm Hg	53.0 (42, 66)	52.5 (43, 67)	54.0 (41, 68)	53.0 (43, 66)	50.5 (38, 60)	0.23
AVA, cm^2^	0.60 ± 0.24	0.64 ± 0.16	0.59 ± 0.19	0.63 ± 0.30	0.72 ± 0.42	0.37
Maximum velocity, m/s	4.75 ± 0.84	4.72 ± 0.54	4.89 ± 0.68	4.87 ± 0.78	4.42 ± 0.83	0.42
LVEF (%)	53.8 ± 13.6	56.1 ± 14.2	53.8 ± 12.5	52.1 ± 13.5	51.5 ± 14.7	0.05
HFpEF (%)	67.0 (207/309)	74.4 (58/78)	70.1 (54/77)	63.6 (49/77)	59.7 (46/77)	0.21
NYHA functional class						
III or IV	89.3 (276/309)	87.2 (68/78)	85.7 (66/77)	93.5 (72/77)	90.9 (70/77)	0.39
PVD	22.7 (70/309)	16.7 (13/78)	20.8 (16/77)	29.9 (23/77)	23.4 (18/77)	0.26
Prior PCI	12.9 (40/309)	10.3 (8/78)	10.4 (8/77)	13.0 (10/77)	18.2 (14/77)	0.42
Prior MI	1.6 (5/309)	0 (0/78)	2.6 (2/77)	1.3 (1/77)	2.6 (2/77)	0.52
Prior CABG	0.3 (1/309)	0 (0/78)	1.3 (1/77)	0 (0/77)	0 (0/77)	0.39
Prior stroke	5.5 (17/309)	3.8 (3/78)	7.8 (6/77)	3.9 (3/77)	6.5 (5/77)	0.63
Smoker	12.3 (38/309)	14.1 (11/78)	9.1 (7/77)	13.0 (10/77)	13.0 (10/77)	0.79
Hypertension	55.0 (170/309)	62.8 (49/78)	41.6 (32/77)	59.7 (46/77)	55.8 (43/77)	0.04
Dyslipidemia	23.3 (72/309)	25.6 (20/78)	20.8 (16/77)	22.1 (17/77)	24.7 (19/77)	0.88
Diabetes mellitus	22.0 (68/309)	24.4 (19/78)	22.1 (17/77)	18.2 (14/77)	23.4 (18/77)	0.80
COPD	22.7 (70/309)	25.6 (20/78)	19.5 (15/77)	23.4 (18/77)	22.1 (17/77)	0.83
NT-proBNP (pg/mL)	2837 (991–8533)	367 (204–742)	1822 (1361–2494)	5178 (3808–6543)	17,427 (12,420–26,013)	< 0.001
Creatinine (μmol/L)	96.9 ± 84.4	70.2 ± 17.2	80.7 ± 18.7	92.0 ± 29.2	125.0 ± 100.2	< 0.001
eGFR (mL/min/1.73m^2^)	70.7 ± 22.9	82.7 ± 16.2	75.6 ± 16.8	68.8 ± 20.4	56.7 ± 24.5	< 0.001

NT-proCNP, N-terminal pro C-type natriuretic peptide; STS, Society of Thoracic Surgeons; TTE, transthoracic echocardiography; AVA, aortic valve area; LVEF, left ventricular ejection fraction; HFpEF, heart failure with preserved EF; PVD, peripheral vascular disease; PCI, percutaneous coronary intervention; MI, myocardial infarction; CABG, coronary artery bypass grafting; COPD, chronic obstructive pulmonary disease; NT-proBNP, N-terminal pro B-type natriuretic peptide; eGFR, estimated glomerular filtration rate

### The predictive capability of each model for cumulative mortality in patients undergoing TAVR

**Fig. 1:**
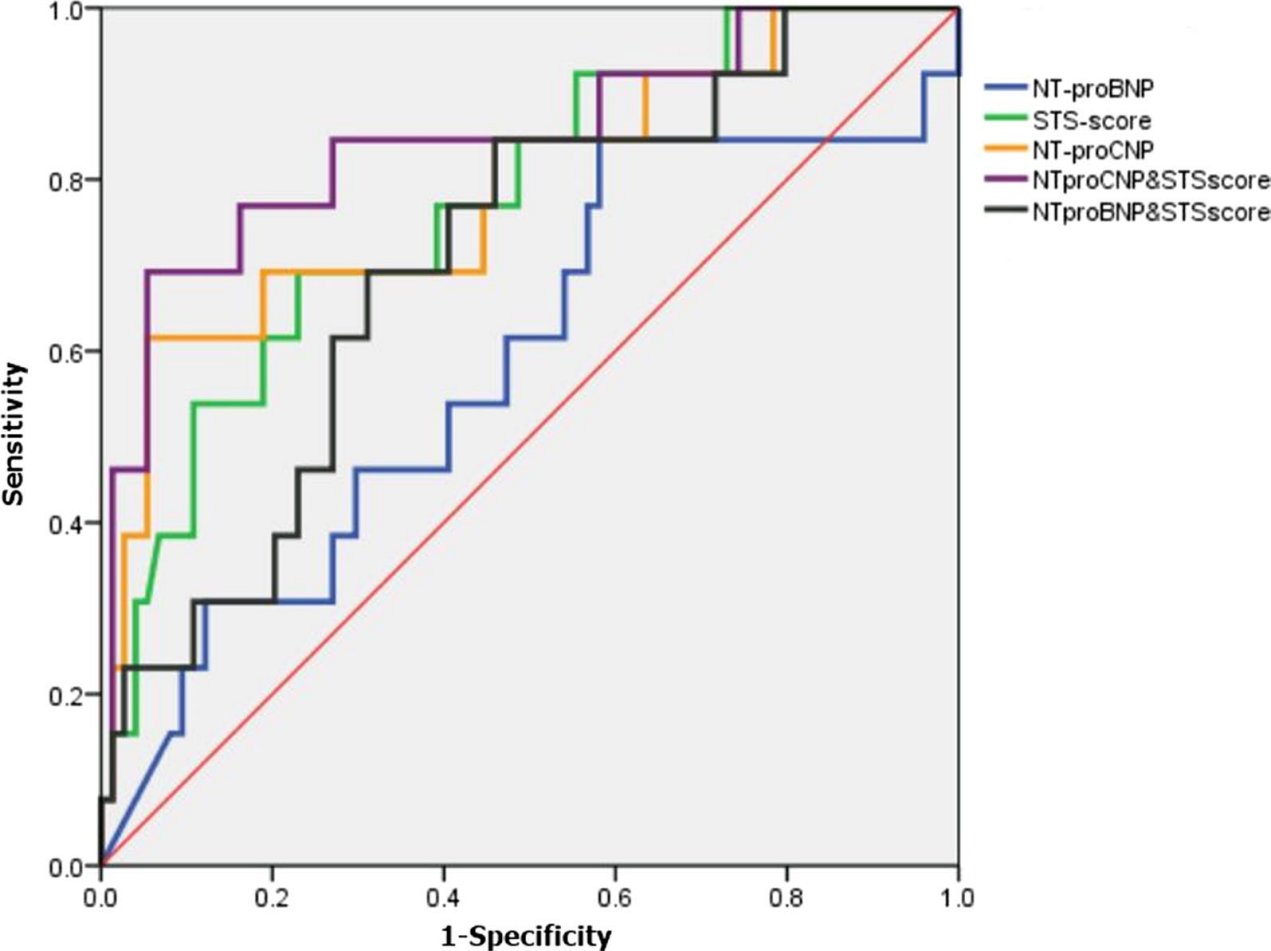
Predictive capability of STS-Score, NT-proBNP, NT-proCNP, combining STS-Score with NT-proCNP/ NT-proBNP for cardiovascular mortality in patients with LVEF < 50%. STS, Society of Thoracic Surgeons; NT-proCNP, N-terminal pro C-type natriuretic peptide; NT-proBNP, N-terminal pro B-type natriuretic peptide

The predictive capability was compared between NT-proBNP and NT-proCNP using receiver operator curve (ROC) analysis. The performances of the models were summarized in Table 2. AUC-values for cumulative mortality were comparable between NT-proBNP and NT-proCNP. ROC analysis yielded AUC-values of 0.79 (0.69; 0.87) for NT-proCNP, confirming relatively high predictive value of cardiovascular mortality in patients with LVEF < 50% (Fig. 1). The comparison of ROC curves indicated that NT-proCNP was superior to NT-proBNP for TAVR risk evaluation in patients with LVEF < 50% [AUC-values of 0.79 (0.69; 0.87) vs. 0.59 (0.48; 0.69), *P* = 0.0453] (Additional file 2: Table 2S). Combining STS-score with NT-proCNP improved predictive ability of cardiovascular mortality in patients with LVEF < 50% compared with STS-score alone, whereas there was no statistical significance (Additional file 2: Table 2S).

**Table 2:** Model performances for potential risk factors of cumulative mortality in 309 patients undergoing transcatheter aortic valve replacement

Variable	*P* value		Model AUC (95% CI)		Hosmer–Lemeshow p		Cut off values	
	All-cause death	Cardiovascular death	All-cause death	Cardiovascular death	All-cause death	Cardiovascular death	All-cause death	Cardiovascular death
STS-Score	< 0.001	< 0.001	0.66 (0.60–0.71)	0.70 (0.65–0.75)	0.79	0.74	9.5	8.8
EF ≥ 50%	0.028	0.01	0.63 (0.56–0.69)	0.66 (0.59–0.73)	0.41	0.19		
EF < 50%	0.01	< 0.001	0.67 (0.57–0.76)	0.77 (0.68–0.85)	0.66	0.90		
NT-ProBNP	< 0.001	0.001	0.67 (0.61–0.72)	0.67 (0.62–0.73)	0.39	0.53	5190	5827
EF ≥ 50%	0.002	< 0.001	0.67 (0.60–0.74)	0.72 (0.65–0.78)	0.94	0.60		
EF < 50%	0.23	0.36	0.59 (0.48–0.69)	0.59 (0.48–0.69)	0.75	0.46		
NT-ProCNP	< 0.001	< 0.001	0.66 (0.60–0.71)	0.74(0.68–0.79)	0.87	0.98	57.8	47.0
EF ≥ 50%	0.031	0.002	0.63 (0.56–0.69)	0.69(0.63–0.76)	0.99	0.56		
EF < 50%	0.005	< 0.001	0.67 (0.57–0.76)	0.79 (0.69; 0.87)	0.94	0.64		
NT-ProBNP + STS-Score	< 0.001	< 0.001	0.68(0.62–0.74)	0.71(0.66–0.77)	0.10	0.03	–	–
EF ≥ 50%	0.003	< 0.001	0.67(0.60–0.74)	0.72(0.65–0.78)	0.10	0.31	–	–
EF < 50%	0.045	0.007	0.64(0.53–0.74)	0.71(0.60–0.80)	0.67	0.67	–	–
NT-ProCNP + STS-Score	< 0.001	< 0.001	0.68(0.62–0.73)	0.75(0.70–0.80)	0.71	0.15	–	–
EF ≥ 50%	0.021	0.001	0.64(0.57–0.70)	0.70(0.63–0.76)	0.32	0.08	–	–
EF < 50%	0.003	< 0.001	0.69(0.59–0.78)	0.85(0.76–0.91)	0.27	0.61	–	–

STS, Society of Thoracic Surgeons; NT-proCNP, N-terminal pro C-type natriuretic peptide; NT-proBNP, N-terminal pro B-type natriuretic peptide; EF, left ventricular ejection fraction

### NT-proCNP levels and outcomes in patients undergoing TAVR

Outcomes-based on baseline quartiles of plasma NT-proCNP are shown in Table 3. No patient was lost to follow-up after a median of 32 months (range 14–48). Among 309 total subjects, 242 (78.3%) patients remained free of major adverse cardiac events, whereas 58 (18.8%) patients died during follow-up. Compared to patients in the first to third quartiles of NT-proCNP, the 1-year cardiovascular mortality rates were significantly higher in the fourth quartile. Notably, the baseline NT-proCNP level in the upper fourth quartile showed a significant association with higher cumulative all-cause and cardiovascular mortality after TAVR (*P* ≤ 0.001).

**Table 3:** Clinical outcomes based on baseline quartiles of NT-proCNP

	Q1 (n = 78)	Q2 (n = 77)	Q3 (n = 77)	Q1 + Q2 + Q3 (n = 232)	Q4 (n = 77)	*P* value
Follow-up time, median (m)	36 (19,45)	27 (13,48)	36 (18,49)	34 (14,48)	28 (13,46)	
1-year						
Mortality, n (%)						
All-cause	5 (6.4)	7 (9.1)	3 (3.9)	15 (6.5)	8 (10.4)	0.138
Cardiovascular	3 (3.8)	4 (5.2)	3 (3.9)	10 (4.3)	9 (11.7)	0.048
MACE, n (%)	6 (7.7)	8 (10.4)	4 (5.2)	18 (7.6)	11 (14.3)	0.089
Cumulative incidence						
Mortality, n (%)						
All-cause	8 (10.3)	13 (16.9)	13 (16.9)	34 (14.7)	24 (31.2)	0.001
Cardiovascular	3 (3.8)	6 (7.8)	8 (10.4)	17 (7.3)	20 (25.8)	< 0.001
MACE, n (%)	10 (12.8)	14 (18.2)	16 (20.8)	40 (17.2)	27 (35.1)	0.001

*P* value: comparing the fourth quartile with the lower three quartiles; MACE, defined as composite of all-cause mortality, myocardial infarction, stroke, permanent pacemaker implantation, new-onset atrial fibrillation, and reoperation for prosthetic valve degeneration

**Fig. 2:**
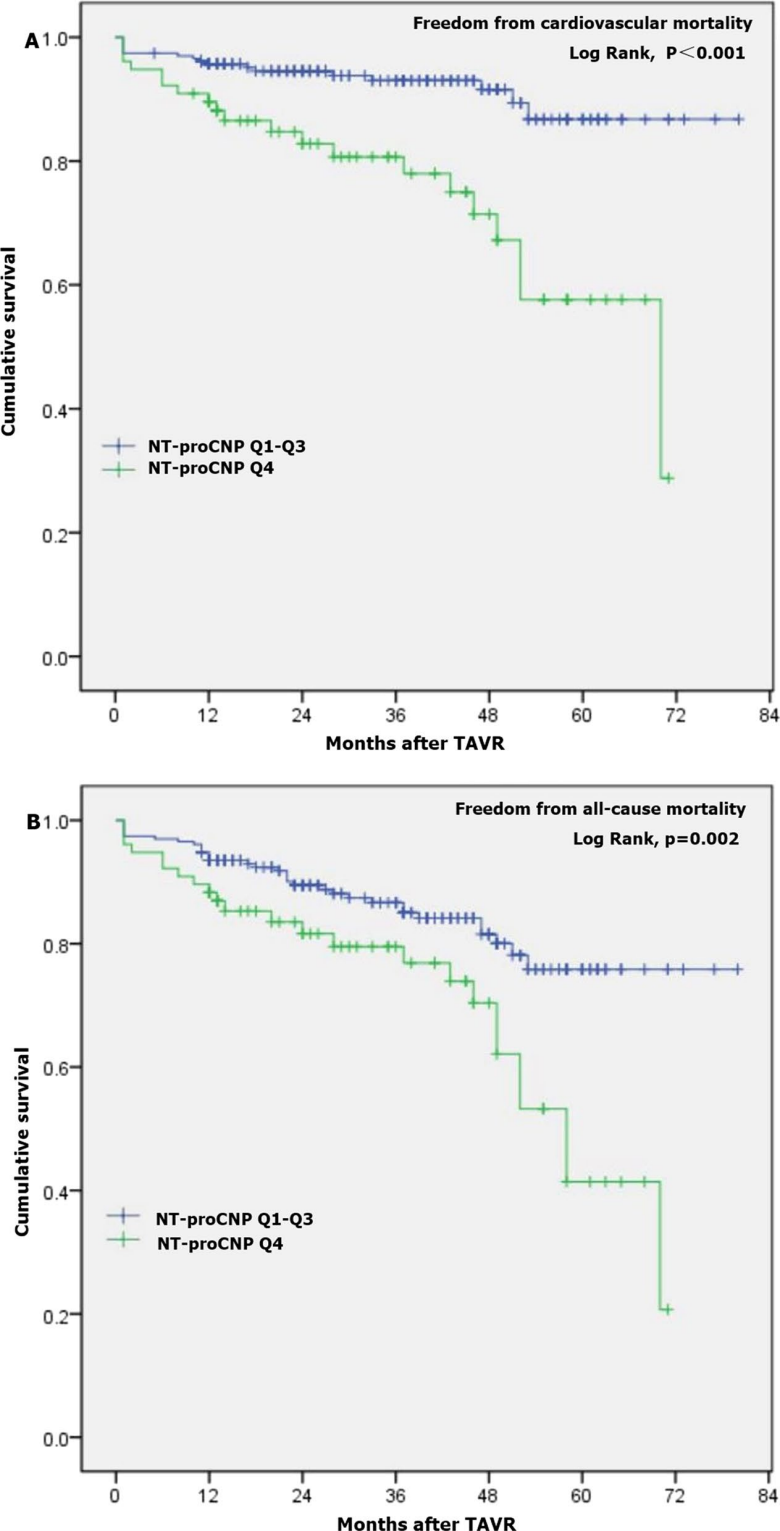
Kaplan–Meier curves for transcatheter aortic valve replacement (TAVR)- related mortality based on NT-proCNP. **A** cardiovascular mortality based on quartiles of NT-proCNP; **B** all-cause mortality based on quartiles of NT-proCNP. NT-proCNP, N-terminal pro C-type natriuretic peptide

The unadjusted Kaplan–Meier estimates of mortality during follow-up were significantly higher in the fourth quartile compared with the first to third quartile (Fig. 2). Our current study has suggested that creatinine is significantly related to NT-proCNP levels; adjustment was made for creatinine instead of eGFR. Cox multivariable analyses indicated STS-Score was a strong independent predictor for cardiovascular mortality (HR 1.10, 95% CI 1.06–1.14, *P* < 0.001) and All-cause mortality (HR 1.08, 95% CI 1.05–1.12, *P* < 0.001) (Table 4). After adjusting for baseline characteristics in the Cox multivariable model, elevated NT-proCNP tended to have higher cardiovascular mortality (HR 1.02, 95% CI 1.00–1.03, *P* = 0.025) and all-cause mortality (HR 1.01, 95% CI 1.00–1.03, *P* = 0.027) (Table 4). However, NT-proBNP showed a small effect size on cardiovascular mortality (HR 1.000054, 95% CI (1.000004–1.000104, P = 0.034) and all-cause mortality (HR 1.000047, 95% CI 1.000009–1.000086, *P* = 0.015) (Additional file 1: Table 1S).

**Table 4:** Cox multivariable models for determinants of cumulative mortality

Tested variables	Cardiovascular mortality		All-cause mortality	
	HR (95% CI)	*P* value	HR (95% CI)	*P* value
Age	0.98 (0.92–1.04)	0.430	1.00 (0.95–1.06)	0.890
Male sex	1.39 (0.63–3.07)	0.409	1.50 (0.80–2.81)	0.210
BMI	0.95 (0.87–1.05)	0.334	0.97 (0.89–1.05)	0.435
Hypertension	1.04 (0.49–2.19)	0.927	0.97 (0.89–1.05)	0.929
LVEF	1.02 (0.99–1.05)	0.222	1.00 (0.98–1.02)	0.875
NT-proCNP	1.02 (1.00–1.03)	0.025	1.01 (1.00–1.03)	0.027
STS score	1.10 (1.06–1.14)	< 0.001	1.08 (1.05–1.12)	< 0.001
Creatinine	1.00 (0.99–1.00)	0.328	1.00 (0.99–1.00)	0.359
Access	0.58 (0.23–1.49)	0.257	0.75 (0.43–1.29)	0.291
Prosthetic valve	1.11 (0.83–1.49)	0.484	1.10 (0.85–1.36)	0.535

HR, hazard ratio; CI, confidential interval

## Discussion

The current study examined the prognostic value of baseline NT-proCNP levels on post-TAVR clinical outcomes and compared its efficacy with NT-proBNP. Our results revealed that baseline NT-proCNP was an independent predictor for mortality, whereas NT-proBNP showed a small effect size on mortality. Furthermore, the capability of NT-proCNP to predict cardiovascular mortality was markedly higher than NT-proBNP in patients with LVEF < 50%.

TAVR has become the alternative treatment for patients with severe symptomatic aortic stenosis [18, 19]. Yet, methods for risk evaluation are essential in reducing TAVR-associated mortality. STS is routinely used to stratify patients into risk levels during the TAVR workup. Our analysis demonstrated that STS-score was a strong independent predictor of mortality for patients undergoing TAVR. Furthermore, biomarkers were useful in cardiovascular disease management, diagnosis, prognostication, and identification of high-risk patients [21]. Increasing evidence suggests that the biomarker-based approach is useful in the risk evaluation of patients undergoing TAVR [22–26].

NT-proBNP and BNP represent established biomarkers for TAVR risk evaluation, valuable in predicting both short- and long-term prognosis [27–29]. Nevertheless, Krau et al., reported that NT-proBNP did not remain significant for mortality in a Cox multivariable model [26]. This was consistent with a study conducted by Seiffert et al., who reported that NT-proBNP could only marginally improve risk evaluation [30]. Our analysis demonstrated that NT-proBNP was poorly predictive of all-cause mortality [AUC-values of 0.67 (0.61–0.72)] and cardiovascular mortality [(AUC-values of 0.67 (0.62–0.73)] in patients undergoing TAVR. NT-proBNP showed a small effect size on mortality in our Cox multivariable model. Interestingly, the comparison of ROC curves indicated that NT-proCNP was superior to NT-proBNP for TAVR risk evaluation in patients with LVEF) < 50% (*P* = 0.0453). Multivariable Cox analyses revealed that increased NT-proCNP was associated with elevated mortality.

The aortic valve opens and closes approximately 3 billion times during a lifetime, making it one of the most mechanically demanding environments in a person's body [31]. Aortic valve stenosis leads to high levels of shear stress at the valve orifice [32] and significantly changes hemodynamics shear stress in the aorta [33]. CNP is also produced in the heart and normal aortic valve, with higher expression in ventricular side valvular endothelial cells [34, 35], which is consistent with it being a shear-sensitive protein [36, 37]. One of the primary hemodynamic forces, shear stress, may be an important physiological regulator of CNP expression [36]. This was supported by Chun et al., who reported sustained augmentation of CNP mRNA expressions in endothelial cells under physiological shear stress [37]. The significant association of CNP/NT-proCNP with heart failure and shear stress might explain why higher NT-proCNP levels are more predictive in TAVR patients compared with NT-proBNP. ROC analysis indicated that predictive values of NT-proCNP were more remarkable than NT-proBNP in patients with LVEF < 50%. One reason may be that the prognosis of heart failure with preserved EF (HFpEF) is even more driven by co-morbidities [14], and another one may be that NT-proCNP levels are significantly related to clinical severity [12].

### Limitations

This study has several limitations. First, although our findings suggested that elevated baseline NT-proCNP had an association with higher cardiovascular mortality after multivariable adjustment, it is not possible to rule out other unmeasured confounders correlated with NT-proCNP. In addition, the relatively small sample may affect an accurate assessment of the value of NT-proCNP for risk evaluation in TAVR patients. Furthermore, the current study was conducted at a single center, which may have led to a selection bias and less generalizable results. Finally, STS is an established predictor for outcome after cardiac surgery and TAVR with well-known cutoff values for low-risk, intermediate-risk, and high-risk patients. The cutoff point of STS in our study only reflects which subgroup of the included patients have a higher risk of mortality after TAVR.

## Conclusions

Baseline NT-proCNP and STS-Score were the independent risk factors of mortality among patients undergoing TAVR. NT-proCNP were superior to NT-proBNP for TAVR risk evaluation, and the advantages were more remarkable in patients with LVEF < 50%. Although NT-proCNP is a better predictor of mortality after TAVR than NT-proBNP, it only showed marginal incremental predictive power beyond STS score. Future multicenter studies with more patients are needed to validate the adverse consequences of elevated baseline NT-proCNP.

## Supplementary Information

**Additional file 1: Table 1S.** Cox multivariable models for determinants of cumulative mortality.

**Additional file 2: Table 2S.** Comparison of ROC curves in patients with LVEF lower than 50%.

## Data Availability

The datasets used and/or analyzed during the current study are available from the corresponding author on reasonable request.

## Abbreviations

NT-proCNP: N-terminal pro C-type natriuretic peptide
STS: Society of thoracic surgeons
TTE: Transthoracic echocardiography
AVA: Aortic valve area
LVEF: Left ventricular ejection fraction
HFpEF: Heart failure with preserved EF
PVD: Peripheral vascular disease
PCI: Percutaneous coronary intervention
MI: Myocardial infarction
CABG: Coronary artery bypass grafting
COPD: Chronic obstructive pulmonary disease
NT-proBNP: N-terminal pro B-type natriuretic peptide
eGFR: Estimated glomerular filtration rate
BMI: Body Mass Index
HR: Hazard ratio
CI: Confidential interval

## References

[1] Sangaralingham SJ, McKie PM, Ichiki T, Scott CG, Heublein DM, Chen HH, Bailey KR, Redfield MM, Rodeheffer RJ, Burnett JC (2015). Circulating C-type natriuretic peptide and its relationship to cardiovascular disease in the general population. Hypertension.

[2] Soeki T, Kishimoto I, Okumura H, Tokudome T, Horio T, Mori K, Kangawa K (2005). C-type natriuretic peptide, a novel antifibrotic and antihypertrophic agent, prevents cardiac remodeling after myocardial infarction. J Am Coll Cardiol.

[3] Sangaralingham SJ, Huntley BK, Martin FL, McKie PM, Bellavia D, Ichiki T, Harders GE, Chen HH, Burnett JC (2011). The aging heart, myocardial fibrosis, and its relationship to circulating C-type natriuretic peptide. Hypertension.

[4] Ichiki T, Schirger JA, Huntley BK, Brozovich FV, Maleszewski JJ, Sandberg SM, Sangaralingham SJ, Park SJ, Burnett JC (2014). Cardiac fibrosis in end-stage human heart failure and the cardiac natriuretic peptide guanylyl cyclase system: regulation and therapeutic implications. J Mol Cell Cardiol.

[5] Horio T, Tokudome T, Maki T, Yoshihara F, Suga S, Nishikimi T, Kojima M, Kawano Y, Kangawa K (2003). Gene expression, secretion, and autocrine action of C-type natriuretic peptide in cultured adult rat cardiac fibroblasts. Endocrinology.

[6] Del Ry S, Passino C, Emdin M, Giannessi D (2006). C-type natriuretic peptide and heart failure. Pharmacol Res.

[7] Peltonen TO, Taskinen P, Soini Y, Rysa J, Ronkainen J, Ohtonen P, Satta J, Juvonen T, Ruskoaho H, Leskinen H (2007). Distinct downregulation of C-type natriuretic peptide system in human aortic valve stenosis. Circulation.

[8] Stingo AJ, Clavell AL, Heublein DM, Wei CM, Pittelkow MR, Burnett JC (1992). Presence of C-type natriuretic peptide in cultured human endothelial cells and plasma. Am J Physiol.

[9] Scotland RS, Ahluwalia A, Hobbs AJ (2005). C-type natriuretic peptide in vascular physiology and disease. Pharmacol Ther.

[10] Vlachopoulos C, Ioakeimidis N, Terentes-Printzios D, Rokkas K, Aznaouridis K, Baou K, Bratsas A, Fassoulakis C, Stefanadis C (2009). Amino-terminal pro-C-type natriuretic peptide is associated with the presence, severity, and duration of vasculogenic erectile dysfunction. Eur Urol.

[11] Prickett TC, Yandle TG, Nicholls MG, Espiner EA, Richards AM (2001). Identification of amino-terminal pro-C-type natriuretic peptide in human plasma. Biochem Biophys Res Commun.

[12] Del Ry S, Cabiati M, Stefano T, Catapano G, Caselli C, Prescimone T, Passino C, Emdin M, Giannessi D (2011). Comparison of NT-proCNP and CNP plasma levels in heart failure, diabetes and cirrhosis patients. Regul Pept.

[13] Jaarsma T, Van Der Wal MH, Hogenhuis J, Lesman I, Luttik ML, Veeger NJ, Van Veldhuisen DJ (2004). Design and methodology of the COACH study: a multicenter randomised coordinating study evaluating outcomes of advising and counselling in heart failure. Eur J Heart Fail.

[14] Lok DJ, Klip IT, Voors AA, Lok SI (2014). Prognostic value of N-terminal pro C-type natriuretic peptide in heart failure patients with preserved and reduced ejection fraction. Eur J Heart Fail.

[15] Nkomo VT, Gardin JM, Skelton TN, Gottdiener JS, Scott CG, Enriquez-Sarano M (2006). Burden of valvular heart diseases: a population-based study. Lancet.

[16] Hu P, Liu XB, Liang J, Zhu QF, Pu CX, Tang MY, Wang JA (2017). A hospital-based survey of patients with severe valvular heart disease in China. Int J Cardiol.

[17] Zaid RR, Barker CM, Little SH, Nagueh SF (2013). Pre- and post-operative diastolic dysfunction in patients with valvular heart disease: diagnosis and therapeutic implications. J Am Coll Cardiol.

[18] Baumgartner H, Falk V, Bax JJ, De Bonis M, Hamm C, Holm PJ, Iung B, Lancellotti P, Lansac E, Rodriguez Muñoz D (2017). 2017 ESC/EACTS Guidelines for the management of valvular heart disease. Eur Heart J.

[19] Otto CM, Nishimura RA, Bonow RO, Carabello BA, Erwin JP, Gentile F, Jneid H, Krieger EV, Mack M, McLeod C (2021). 2020 ACC/AHA guideline for the management of patients with valvular heart disease: a report of the American College of Cardiology/American Heart Association Joint Committee on Clinical Practice Guidelines. Circulation.

[20] Fan J, Fang X, Liu C, Zhu G, Hou CR, Jiang J, Lin X, Wang L, He Y, Zhu Q (2020). Brain injury after transcatheter replacement of bicuspid versus tricuspid aortic valves. J Am Coll Cardiol.

[21] Matusik PT (2019). Biomarkers and cardiovascular risk stratification. Eur Heart J.

[22] Aung N, Dworakowski R, Byrne J, Alcock E, Deshpande R, Rajagopal K, Brickham B, Monaghan MJ, Okonko DO, Wendler O (2013). Progressive rise in red cell distribution width is associated with poor outcome after transcatheter aortic valve implantation. Heart.

[23] Gotzmann M, Thiessen A, Lindstaedt M, Mügge A, Ewers A (2013). Left atrial diameter, aortic mean gradient, and hemoglobin for risk stratification in patients undergoing transcatheter aortic valve implantation. Clin Cardiol.

[24] Yamamoto M, Hayashida K, Mouillet G, Hovasse T, Chevalier B, Oguri A, Watanabe Y, Dubois-Randé JL, Morice MC, Lefèvre T (2013). Prognostic value of chronic kidney disease after transcatheter aortic valve implantation. J Am Coll Cardiol.

[25] Frank D, Stark S, Lutz M, Weissbrodt A, Freitag-Wolf S, Petzina R, Rosenberg M, Lutter G, Frey N (2013). Preprocedural high-sensitive troponin predicts survival after transcatheter aortic valve implantation (TAVI). Int J Cardiol.

[26] Krau NC, Lünstedt NS, Freitag-Wolf S, Brehm D, Petzina R, Lutter G, Bramlage P, Dempfle A, Frey N, Frank D (2015). Elevated growth differentiation factor 15 levels predict outcome in patients undergoing transcatheter aortic valve implantation. Eur J Heart Fail.

[27] López-Otero D, Trillo-Nouche R, Gude F, Cid-Álvarez B, Ocaranza-Sanchez R, Alvarez MS, Lear PV, Gonzalez-Juanatey JR (2013). Pro B-type natriuretic peptide plasma value: a new criterion for the prediction of short- and long-term outcomes after transcatheter aortic valve implantation. Int J Cardiol.

[28] Chen S, Redfors B, O'Neill BP, Clavel MA, Pibarot P, Elmariah S, Nazif T, Crowley A, Ben-Yehuda O, Finn MT (2020). Low and elevated B-type natriuretic peptide levels are associated with increased mortality in patients with preserved ejection fraction undergoing transcatheter aortic valve replacement: an analysis of the PARTNER II trial and registry. Eur Heart J.

[29] O'Leary JM, Clavel MA, Chen S, Goel K, O'Neill B, Elmariah S, Crowley A, Alu MC, Thourani VH, Leon MB (2020). Association of natriuretic peptide levels after transcatheter aortic valve replacement with subsequent clinical outcomes. JAMA Cardiol.

[30] Seiffert M, Sinning JM, Meyer A, Wilde S, Conradi L, Vasa-Nicotera M, Ghanem A, Kempfert J, Hammerstingl C, Ojeda FM (2014). Development of a risk score for outcome after transcatheter aortic valve implantation. Clin Res Cardiol Off J German Cardiac Soc.

[31] Gould ST, Srigunapalan S, Simmons CA, Anseth KS (2013). Hemodynamic and cellular response feedback in calcific aortic valve disease. Circ Res.

[32] Baratchi S, Zaldivia MTK, Wallert M, Loseff-Silver J, Al-Aryahi S, Zamani J, Thurgood P, Salim A, Htun NM, Stub D (2020). Transcatheter aortic valve implantation represents an anti-inflammatory therapy via reduction of shear stress-induced, piezo-1-mediated monocyte activation. Circulation.

[33] van Ooij P, Markl M, Collins JD, Carr JC, Rigsby C, Bonow RO, Malaisrie SC, McCarthy PM, Fedak PWM, Barker AJ (2017). Aortic valve stenosis alters expression of regional aortic wall shear stress: new insights from a 4-dimensional flow magnetic resonance imaging study of 571 subjects. J Am Heart Assoc.

[34] Simmons CA, Grant GR, Manduchi E, Davies PF (2005). Spatial heterogeneity of endothelial phenotypes correlates with side-specific vulnerability to calcification in normal porcine aortic valves. Circ Res.

[35] Palmer SC, Prickett TC, Espiner EA, Yandle TG, Richards AM (2009). Regional release and clearance of C-type natriuretic peptides in the human circulation and relation to cardiac function. Hypertension.

[36] Zhang Z, Xiao Z, Diamond SL (1999). Shear stress induction of C-type natriuretic peptide (CNP) in endothelial cells is independent of NO autocrine signaling. Ann Biomed Eng.

[37] Chun TH, Itoh H, Ogawa Y, Tamura N, Takaya K, Igaki T, Yamashita J, Doi K, Inoue M, Masatsugu K (1997). Shear stress augments expression of C-type natriuretic peptide and adrenomedullin. Hypertension.

