# Supplementary material for: Elevated N-terminal pro C-type natriuretic peptide is associated with mortality in patients undergoing transcatheter aortic valve replacement
Source: BMC Cardiovasc Disord. 2022 Apr 12;22:164. doi: 10.1186/s12872-022-02615-8 (PMC9004019; doi:10.1186/s12872-022-02615-8)
Supplement: Supplementary file 1 — Additional file 1: Table 1S. Cox multivariable models for determinants of cumulative mortality. [file 12872_2022_2615_MOESM1_ESM.docx]

Table 1S Cox multivariable models for determinants of cumulative mortality

|  | Cardiovascular mortality | | All-cause mortality | |
| --- | --- | --- | --- | --- |
| Tested variables | HR (95% CI) | P-value | HR (95% CI) | P-value |
| Age | 0.98 (0.92-1.05) | 0.607 | 1.01 (0.95-1.06) | 0.829 |
| Male sex | 1.96 (0.86-4.49) | 0.110 | 2.00 (1.02-3.90) | 0.043 |
| BMI | 0.98 (0.89-1.09) | 0.754 | 1.00 (0.92-1.09) | 0.995 |
| Hypertension | 1.14 (0.52-2.48) | 0.750 | 1.00 (0.54-1.87) | 0.996 |
| LVEF | 1.04 (1.00-1.07) | 0.069 | 1.02 (0.99-1.05) | 0.180 |
| NT-proBNP | 1.000054  (1.000004-1.000104) | 0.034 | 1.000047  (1.000009-1.000086) | 0.015 |
| STS score | 1.08 (1.04-1.12) | <0.001 | 1.07 (1.04-1.11) | <0.001 |
| Creatinine | 1.00 (1.00-1.01) | 0.127 | 1.00 (1.00-1.00) | 0.107 |
| Access | 0.61 (0.24-1.57) | 0.301 | 0.79 (0.46-1.37) | 0.406 |
| Prosthetic valve | 1.06 (0.78-1.43) | 0.729 | 1.04 (0.80-1.33) | 0.791 |

HR, hazard ratio; CI, confidential interval.
