# Supplementary material for: Elevated N-terminal pro C-type natriuretic peptide is associated with mortality in patients undergoing transcatheter aortic valve replacement
Source: BMC Cardiovasc Disord. 2022 Apr 12;22:164. doi: 10.1186/s12872-022-02615-8 (PMC9004019; doi:10.1186/s12872-022-02615-8)
Supplement: Supplementary file 2 [file 12872_2022_2615_MOESM2_ESM.doc]

Comparison of ROC curves




# Comparison of ROC curves in patients with LVEF lower than 50%

## Pairwise comparison of ROC curves

|  |  |
| --- | --- |
| NTproBNP ~ NTproCNP | |
| Difference between areas | 0.203 |
| Standard Error ^a^ | 0.101 |
| 95% Confidence Interval | 0.00420 to 0.401 |
| z statistic | 2.001 |
| Significance level | P = 0.0453 |
| NTproBNP ~ STS\_score | |
| Difference between areas | 0.188 |
| Standard Error ^a^ | 0.0982 |
| 95% Confidence Interval | -0.00488 to 0.380 |
| z statistic | 1.910 |
| Significance level | P = 0.0561 |
| NTproCNP ~ STS\_score | |
| Difference between areas | 0.0151 |
| Standard Error ^a^ | 0.0920 |
| 95% Confidence Interval | -0.165 to 0.195 |
| z statistic | 0.164 |
| Significance level | P = 0.8699 |
| STS\_score ~ NTproBNP&STS score | |
| Difference between areas | 0.0660 |
| Standard Error ^a^ | 0.0453 |
| 95% Confidence Interval | -0.0228 to 0.155 |
| z statistic | 1.457 |
| Significance level | P = 0.1452 |
| STS\_score ~ NTproCNP&STS score | |
| Difference between areas | 0.0743 |
| Standard Error ^a^ | 0.0617 |
| 95% Confidence Interval | -0.0466 to 0.195 |
| z statistic | 1.205 |
| Significance level | P = 0.2282 |

 ^a^ DeLong et
al., 1988
